# Roles of Gut-Derived Secretory Factors in the Pathogenesis of Non-Alcoholic Fatty Liver Disease and Their Possible Clinical Applications

**DOI:** 10.3390/ijms19103064

**Published:** 2018-10-08

**Authors:** Hirofumi Okubo, Akifumi Kushiyama, Yusuke Nakatsu, Takeshi Yamamotoya, Yasuka Matsunaga, Midori Fujishiro, Hideyuki Sakoda, Haruya Ohno, Masayasu Yoneda, Tomoichiro Asano

**Affiliations:** 1 Department of Molecular and Internal Medicine, Graduate School of Biomedical and Health Sciences, Hiroshima University, Hiroshima 734-8551, Japan; d7276@hiroshima-u.ac.jp (H.O.); haruyan711@gmail.com (H.O.); 2 Division of Diabetes and Metabolism, The Institute for Adult Diseases, Asahi Life Foundation, Tokyo 103-0002, Japan; a-kushiyama@asahi-life.or.jp; 3 Department of Medical Chemistry, Division of Molecular Medical Science, Graduate School of Biomedical Sciences, Hiroshima University, Hiroshima 734-8551, Japan; nakatsu@hiroshima-u.ac.jp (Y.N.); ymmty@hiroshima-u.ac.jp (T.Y.); 4 Center for Translational Research in Infection & Inflammation, School of Medicine, Tulane University, 6823 St. Charles Avenue, New Orleans, LA 70118, USA; ymatsunaga@tulane.edu; 5 Division of Diabetes and Metabolic Diseases, Nihon University School of Medicine, Tokyo 173-8610, Japan; midori-tky@umin.ac.jp; 6 Division of Neurology, Respirology, Endocrinology and Metabolism, Department of Internal Medicine, Faculty of Medicine, University of Miyazaki, Miyazaki 889-1692, Japan; hsakoda-tky@umin.ac.jp; 7 Department of Preventive Medicine for Diabetes and Lifestyle-related Diseases, Graduate School of Biomedical and Health Sciences, Hiroshima University, Hiroshima 734-8551, Japan; masayone17@hiroshima-u.ac.jp

**Keywords:** non-alcoholic fatty liver disease, non-alcoholic steatohepatitis, gut-liver axis, resistin like molecule β, glucagon-like peptide-1, glucagon-like peptide-2, fibroblast growth factor 19, neurotensin

## Abstract

The rising prevalence of non-alcoholic fatty liver disease (NAFLD) parallels the global increase in the number of people diagnosed with obesity and metabolic syndrome. The gut-liver axis (GLA) plays an important role in the pathogenesis of NAFLD/non-alcoholic steatohepatitis (NASH). In this review, we discuss the clinical significance and underlying mechanisms of action of gut-derived secretory factors in NAFLD/NASH, focusing on recent human studies. Several studies have identified potential causal associations between gut-derived secretory factors and NAFLD/NASH, as well as the underlying mechanisms. The effects of gut-derived hormone-associated drugs, such as glucagon-like peptide-1 analog and recombinant variant of fibroblast growth factor 19, and other new treatment strategies for NAFLD/NASH have also been reported. A growing body of evidence highlights the role of GLA in the pathogenesis of NAFLD/NASH. Larger and longitudinal studies as well as translational research are expected to provide additional insights into the role of gut-derived secretory factors in the pathogenesis of NAFLD/NASH, possibly providing novel markers and therapeutic targets in patients with NAFLD/NASH.

## 1. Introduction

The rising prevalence of non-alcoholic fatty liver disease (NAFLD) parallels the global increase in the number of people diagnosed with obesity and metabolic syndrome. A subset of individuals with NAFLD develops non-alcoholic steatohepatitis (NASH), which is characterized by hepatocellular lipid accumulation along with inflammation and fibrosis of varying severities [1,2,3]. NASH can lead to cirrhosis and even liver cancer. Furthermore, NAFLD is also recognized as an independent risk factor for cardiovascular mortality [4], such that the development of effective therapeutic interventions is eagerly awaited. Despite the very high number of pharmacological interventions currently being proposed, the identification of an effective therapy for NAFLD beyond the widely recommended lifestyle measures remains an open issue and represents a major clinical and research challenge [5].

The strong anatomical and functional interactions between the gastrointestinal tract and the liver underlie the gut-liver axis (GLA) concept. This intimate connection begins in embryogenesis, as the liver and gastrointestinal tract both arise from the ventral foregut endoderm [6]. The GLA is characterized as showing bidirectional traffic. Nutrients and factors derived from the gut lumen reach the liver via the portal circulation; bile acids produced by hepatocytes are then released via the biliary tract into the small intestine. However, this description is overly simple because the GLA has functions beyond its nutritional component. This is a complex axis and alterations, especially in two of its components (intestinal barrier and gut microbiota) appear to play key roles in the onset and progression of liver damage [7]. 

The GLA plays an important role in the pathogenesis of NAFLD/NASH. Recently, we demonstrated possible causal links between gut-derived resistin like molecule β (RELMβ) and NASH, as well as the underlying mechanisms participating in its pathogenesis [8]. The effects of gut-derived hormone-associated drugs for treating NAFLD/NASH have also been reported. Therefore, gut-derived secretory factors represent a novel marker and potential therapeutic target in patients with NAFLD/NASH. 

In this review, we discuss the clinical significance and underlying mechanisms of action of gut-derived secretory factors in NAFLD/NASH.

## 2. RELMβ

RELMβ, a protein homologous to resistin, is expressed in the secretory granules of intestinal goblet cells, macrophages and bronchi [9,10]. Three resistin-related proteins, designated RELM α, β and γ (encoded by the genes *Retnla*, *Retnlb* and *Retnlg*, respectively) have been identified in mice. Resistin and RELMβ have been identified in humans, while RELMα and γ have not [11,12]. 

RELMβ is a bactericidal protein secreted apically into the intestinal lumen which directly kills pathogens [13,14]. Colonic RELMβ expression is undetectably low in germ-free mice [9], and the absence of RELMβ influences the microbial composition in the gut [15]. A recent study demonstrated both mouse and human RELMβ to selectively kill gram-negative bacteria by forming size-selective pores which result in permeabilization of the bacterial membrane [14]. RELMβ also contributes to host defense against intestinal nematode infections. RELMβ is induced by intestinal nematode infections through a host Th2-mediated immune responses, and inhibits both chemotaxis and feeding [16,17]. 

RELMβ also contributes to immune regulation in the gut. RELMβ expression is induced in some animal models of intestinal inflammation [18,19]. RELMβ was considered to be delivered from the lumen into the lamina propria through a disrupted epithelial barrier and to influence macrophage activation resulting in the production of inflammatory cytokines [20]. Indeed, RELMβ knock-out (KO) mice showed suppression of dextran sodium sulfate-induced colitis [20] and infection-induced intestinal inflammation [21]. The RELMβ promoter contains functional binding sites for the hepatocyte nuclear factor 4α (HNF4α), a transcriptional factor expressed in the intestine. HNF4α has two isoforms, P1 and P2, and specific expression of P2-HNF4α in mice promoted RELMβ expression and inflammation in a murine colitis model [22]. RELMβ also reportedly promotes colitis in mucin KO mice with dysbiosis, as evidenced by depleting protective commensal microbiota (*Lactobacillus* spp.). Interestingly, administration of *Lactobacillus* reduced RELMβ positive cells in the mouse colon [23]. Thus, it appears that RELMβ and the gut microbiota regulate each other, with both contributing to the maintenance of gut homeostasis, including immune and inflammatory responses. 

**Figure 1:**
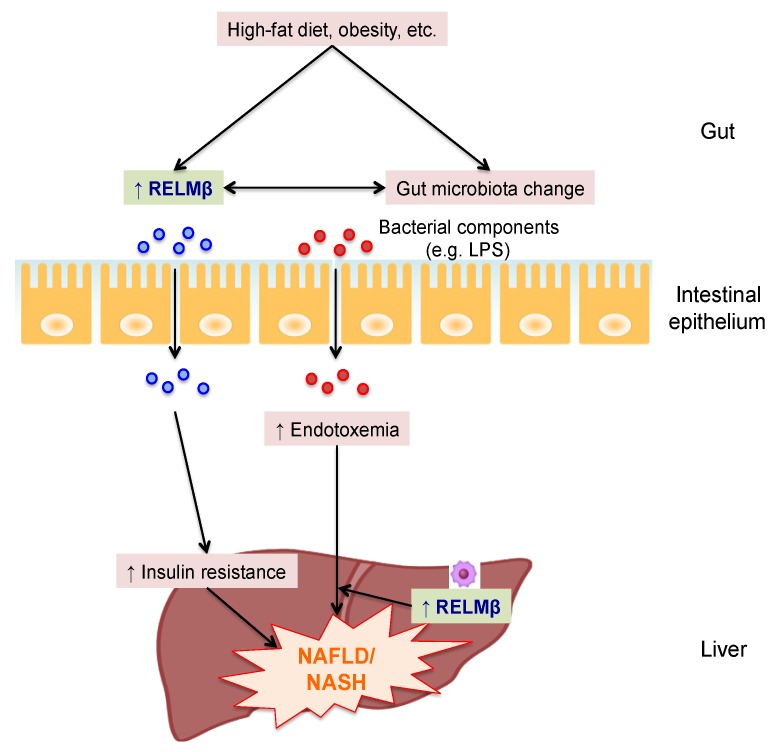
The proposed role of RELMβ in the pathogenesis of NAFLD/NASH. Intake of a high-fat diet and obesity increase both intestinal expression and circulating levels of resistin like molecule β (RELMβ). Circulating RELMβ elicits insulin resistance, and increased gut-derived RELMβ and gut microbiota appear to regulate each other, thereby increasing translocation of the endotoxin lipopolysaccharide (LPS) from the intestine into the bloodstream and liver, which induces hepatic steatosis and inflammation. In the liver, RELMβ in Kupffer cells exacerbates hepatic inflammation, along with endotoxemia, which further worsens non-alcoholic fatty liver disease (NAFLD)/non-alcoholic steatohepatitis (NASH).

Insulin resistance plays an important role in the pathogenesis of NAFLD [24]. In insulin resistant models such as high fat diet (HFD) fed and *db/db* mice, the intestinal expression and serum concentration of RELMβ were both increased [11]. Peripheral infusion of RELMβ impaired insulin action in the rat liver [25]. In addition, transgenic mice overexpressing RELMβ in the liver showed insulin resistance and hepatic steatosis as compared to control mice under HFD-fed conditions. RELMβ contributes to metabolic dysfunction by signaling through mitogen-activated protein kinase pathways and suppressing insulin signaling in hepatocytes [26]. Thus, insulin resistance caused by RELMβ might be involved in the pathogenesis of NAFLD. However, RELMβ KO mice are also resistant to NASH with a higher proportion of lactic acid bacteria (*Lactobacillus* species) in feces and reduced endotoxemia in a methionine choline-deficient (MCD) diet model showing neither obesity nor insulin resistance [8]. The MCD diet was found to increase RELMβ expression in both Kupffer cells (resident macrophages in the liver) and the colon. Interestingly, mouse experiments using radiation chimeras showed wild type and RELMβ KO to be required in both organs for full manifestation of NASH. The RELMβ in Kupffer cells enhanced hepatic inflammation induced by the endotoxin lipopolysaccharide (LPS) entering the liver, since LPS-induced inflammatory cytokine expressions were significantly suppressed in peritoneal macrophages isolated from RELMβ KO mice and a previous report showed RELMβ activated macrophages to express MHC class II and inflammatory cytokines [21]. Regarding the role of RELMβ in the colon, changes in RELMβ-induced gut microbiota might be involved in the impairment of gut permeability and the induction of endotoxemia, since RELMβ KO mice showed a higher proportion of *Lactobacillus*, which reportedly contributes to the normalization of tight junction proteins and reduced endotoxemia [27,28,29]. Though RELMβ itself might be a key factor impairing gut barrier function independently of the gut microbiota, its impact on barrier function is still debated [20,30]. Thus, RELMβ may raise not only the level of circulating LPS but also activate macrophages to be highly responsive to LPS. The proposed role of RELMβ in the pathogenesis of NAFLD/NASH is shown in Figure 1.

A human study demonstrated plasma levels of RELMβ to be measurable [31]. Though further investigation of the relationships of RELMβ with metabolic parameters in humans is needed, the data accumulated thus far suggest that suppressing gut-derived RELMβ expression by approaches such as gut microbiota modification might be a therapeutic strategy for managing NAFLD/NASH. In addition, RELMβ receptors have not as yet been identified. Further investigation of the receptors and activation mechanisms for RELMβ might lead to a new class of agents that antagonize the pathological effects of RELMβ, and could serve as novel therapies for NAFLD/NASH.

## 3. Glucagon-Like Peptide-1

Glucagon-like peptide-1 (GLP-1), a 30-amino acid peptide produced from the proglucagon gene in endocrine L-cells of the small intestine, is secreted into the bloodstream in response to nutrients [32,33]. GLP-1 receptor (GLP-1R) is a G protein coupled receptor widely expressed in pancreatic islets, the stomach, duodenum, central nervous system (CNS), heart, lung, kidney and immune cells [34,35]. Whether GLP-1R is expressed in hepatocytes remains controversial [36,37]. 

GLP-1 reduces blood glucose concentrations by glucose-dependently stimulating insulin secretion and suppressing glucagon secretion, thereby promoting satiety and delaying gastric emptying through central mechanisms, which in turn reduces food intake [38,39]. GLP-1 was also reported to directly reduce steatosis by decreasing lipogenesis and increasing fatty acid oxidation in hepatocytes [36,40,41]. Considering the association of NAFLD/NASH with obesity and type 2 diabetes mellitus (T2DM), these pleiotropic effects make GLP-1 an attractive therapeutic target in patients with NAFLD/NASH. 

The half-life of GLP-1 is short, only one to two minutes, due to N-terminal degradation by the enzyme dipeptidyl peptidase-4 (DPP-4). Synthetic GLP-1R agonists (GLP-1RA) which are resistant to degradation by DPP-4, and DPP-4 inhibitors which prevent GLP-1 degradation, thereby increasing the plasma levels of endogenous GLP-1, are now in widespread clinical use for T2DM [42,43]. Interestingly, glucose-induced GLP-1 secretion is reportedly decreased in patients with NAFLD/NASH as compared to healthy control subjects [44]. In a meta-analysis of 6 randomized-controlled trials, the LEAD (Liraglutide Efficacy and Action in Diabetes) trials, daily injection of GLP-1RA liraglutide (1.8 mg per day) produced a significant decrease in plasma alanine aminotransferase (ALT) (a liver injury biomarker) in patients with T2DM, and the effect was dose-dependent (no significant difference vs. placebo with liraglutide 0.6 or 1.2 mg). This effect appeared to be mediated by GLP-1RA actions on weight loss and glycemic control [45]. Another report showed a significant reduction in liver fat in T2DM patients treated with either the GLP-1RA exenatide or liraglutide for 6 months that correlated with improved glycemic control [46]. The LEAN trial (Liraglutide Efficacy and Action in NASH) was the first randomized, placebo-controlled trial to examine the effect of liraglutide on liver histology in patients with NASH. Participants in the Liraglutide (1.8 mg per day) treatment group showed resolution of definite NASH as compared with participants in the placebo group, although no anti-fibrotic effects were seen. The effects of liraglutide were attributed to a combination of direct hepatic effects and an effect on weight loss. Liraglutide was safe, well tolerated and had an adverse effect profile similar to that of the placebo, with the exception of predictable gastrointestinal symptoms including diarrhea, constipation and loss of appetite [47]. Japanese studies (LEAN J study) also reported that liraglutide (0.9 mg per day) decreased liver fat deposition and improved ALT and aspartate aminotransferase (AST) in patients with NASH [48]. The effects of exenatide in NASH have also been explored (NCT01208649). Semaglutide is a GLP-1 agonist that requires only weekly dosing. In the SUSTAIN 1 trial, drug-naive patients with T2DM were randomly assigned to once weekly semaglutide (0.5 mg or 1.0 mg) or a placebo for 30 weeks. HbA1c, body weight, body mass index (BMI) and waist circumstance were significantly reduced in both semaglutide groups as compared to the placebo group [49]. A large multicenter trial designed to compare the efficacy and safety profiles of three doses of once daily subcutaneous semaglutide versus a placebo in patients with biopsy proven NASH is currently underway (NCT02970942). In addition, oral semaglutide is now being examined in clinical trials [50]. Considering the improved acceptance and adherence, oral semaglutide might be a promising treatment for NASH. However, the American Association for the Study of Liver Diseases warns that it is premature to consider using GLP-1RA to specifically treat NASH/NAFLD in the absence of diabetes because clinical evidence of efficacy and safety is still insufficient [51]. Larger long-term randomized-controlled trials are needed to establish the role of GLP-1RA in the management of NAFLD/NASH patients.

In randomized double-blind placebo-controlled studies, the DPP-4 inhibitor sitagliptin (100 mg per day) was no more effective than a placebo for ameliorating hepatic steatosis and fibrosis in patients with NAFLD/NASH [52,53]. Considering that DPP-4 inhibitors increase endogenous GLP-1 to physiological levels (10–25 pmol/L), whereas GLP-1RA reaches higher pharmacological levels (60–90 pmol/L), the difference in elevated GLP-1 concentrations might contribute to the differing efficacies in patients with NAFLD/NASH, though whether tissue distributions are similar for all GLP-1R and what level of GLP-1 exposure produces each effect, are not well known [43]. The clinical trials involving NAFLD/NASH patients employing GLP-1RA or DPP-4 inhibitors are summarized in Table 1.

A recent study showed HFD-induced dysbiosis to impair GLP-1 signaling involved in the control of insulin secretion and gastric emptying in mice [54]. NAFLD/NASH patients reportedly have dysbiosis [55]. Future studies of GLP-1 resistance in human subjects targeting GLP-1 signaling may reveal whether correcting dysbiosis is a potentially novel strategy for treating NAFLD/NASH.

## 4. Glucagon-Like Peptide-2

Glucagon-like peptide 2 (GLP-2) is a 33-amino acid proglucagon-derived peptide produced by L-cells in the small and large intestine, as well as in the brain, predominantly the brainstem [56]. Prohormone convertase 1/3 processes proglucagon resulting in GLP-1, GLP-2, intervening peptide-2, oxynthomodulin and glicentin in the gastrointestinal tract and in the brain [57].

GLP-2 is secreted in response to nutrients such as carbohydrates and fats [58]. In humans, GLP-2 has a short half-life of about 7 min due to degradation to inactive GLP-2 (3–33) by DPP-IV [59]. DPP-IV-resistant GLP-2 analogues such as teduglutide, in which alanine is replaced with glycine at the second position from the N-terminus of GLP-2, exhibit greater bioactivity relative to the native molecule, due to their longer circulating half-lives [60]. GLP-2 exerts its action by binding the GLP-2 receptor (GLP-2R), a member of the G protein coupled receptor superfamily. GLP-2R exhibits high homology with GLP-1R, with which it shares several intercellular signaling mediators such as cyclic adenosine monophosphate (cAMP) and protein kinase A. GLP-2R is widely expressed in the gastrointestinal tract, CNS, lung, vagal afferents, heart and liver [61,62,63,64,65,66].

GLP-2 is produced by neurons in the brainstem and fibers that project to the dorsomedial hypothalamic nucleus where GLP-2R is located. Intra-cerebroventricular infusion of GLP-2 inhibits food intake in rats. Thus, GLP-2 is considered to be a neurotransmitter which is involved in the control of feeding behavior [67]. While human studies have not demonstrated a decrease in food intake after peripheral GLP-2 administration, the data obtained to date suggest that circulating GLP-2 at physiological concentrations might not be involved in the control of feeding behavior in humans [68].

GLP-2 has also been shown to induce crypt cell proliferation and to inhibit apoptosis, thereby increasing villus height and expanding the absorptive mucosal surface in both the small and the large intestine [56,69,70]. In addition, in parenterally fed rats with short bowel syndrome (SBS), GLP-2 treatment reversed the associated increases in villus height and the intestinal mucosal surface area [71]. Indeed, a GLP-2R agonist, teduglutide, is under clinical development for patients with SBS [72,73].

Energy absorption in the gastrointestinal tract is promoted by GLP2, via mechanisms involving both non-specific and specific adaptation. GLP2 increases the uptakes of sugars via augmented activities and expressions of transporters [74,75], as well as by enhancing the expressions of various digestive enzymes [76]. GLP2 is also involved in lipid absorption. Indeed, GLP2 reportedly facilitates intestinal absorption of lipids [77] and enhances and regulates chylomicron secretion from the intestine in human subjects [78].

GLP-2 modulates intestinal permeability. GLP-2 treated mice show intestinal epithelial barrier enhancement with increased total mucosal thickness and villus height [79]. GLP-2 was also reported to directly enhance intestinal permeability in human intestinal epithelial cells [80]. Pharmacological GLP-2 treatment improves gut permeability markers, while reducing endotoxemia and systemic and hepatic inflammation in genetically obese (*ob/ob*) mice [81]. Patients with NAFLD/NASH reportedly show increased gut permeability and elevated plasma endotoxin concentrations, which are considered to be involved in the pathogenesis of NAFLD/NASH [82,83]. GLP-2 enhances epithelial barrier function and ameliorates inflammation [84], thereby possibly contributing to suppression of the development of NAFLD/NASH. Chronic treatment with GLP-2 (3–33), a competitive GLP-2R antagonist [85], also reportedly exacerbates dyslipidemia and hepatic lipid accumulation in HFD-fed mice [86], suggesting that endogenous GLP-2 may have a defensive role acting against lipid imbalance under conditions of obesity.

At present, commercial GLP-2 measurement kits detect both the 1–33 (active) and 3–33 (inactive) forms of GLP-2 [87,88]. Development of a measurement kit that can detect only the active form of GLP-2, such as is the case for GLP-1, might deepen our understanding of the relationship between GLP-2 and NAFLD/NASH in humans. Furthermore, GLP-2R exerts its actions in the liver. Thus, we need to elucidate the direct effects of GLP-2 in order to understand the role of GLP-2 in the pathogenesis of NAFLD/NASH. Though there are concerns about the potential for carcinogenesis with the use of GLP-2 or its analogues [89] and further studies are needed, GLP2 analogues such as teduglutide might be an attractive approach for clinical treatment of NAFLD/NASH through structural and functional restoration of the intestinal epithelium, which would presumably reduce both intestinal permeability and endotoxemia.

## 5. Fibroblast Growth Factor 19

Fibroblast growth factor (FGF) 15 (in mice)/19 (in humans) is an endocrine gastrointestinal hormone expressed in ileal enterocytes, where it was shown to be induced by the bile acid acting farnesoid X receptor (FXR) [90]. Recent studies have suggested that FGF19 is also regulated by other food-derived components such as fat-soluble vitamins [91] and cholesterol [92].

FGF15/19 binds to its preferred receptor, FGF receptor (FGFR) 4, and co-receptor β-klotho [93]. The liver is the major site of the metabolic actions of FGF19, which are exerted via activation of the FGFR4-β-klotho complex. FGF19 also has biological functions in other tissues such as white adipose tissue, where it binds and activates FGFR1c-β-klotho [93], and in the brain via an as yet unknown FGFR complex [94].

FGF15/19 plays a key role in bile acid- and FXR-mediated CYP7A1 inhibition by binding to the FGFR4-β-klotho complex [90]. Indeed, FGF15, FGFR4 and β-klotho KO animal models all show dysregulated bile acid metabolism, and administration of exogenous FGF19 fails to suppress CYP7A1 in both FGFR4 and β-klotho KO animals [90,95,96,97,98].

In addition to their roles in the regulation of bile acid homeostasis, both FGF15/19 and FGFR4 are involved in the maintenance of glucose and protein metabolism [99]. FGF15 KO mice showed failure of proper blood glucose level maintenance and exhibited reduced hepatic glycogen and glucose intolerance that were corrected by FGF19 administration [100]. The ability of FGF19 to maintain glucose homeostasis also relies on gluconeogenesis inhibition through a pathway involving inhibition of the cAMP response element binding protein—peroxisome proliferator activated receptor-γ co-activator 1α signaling cascade [101]. Conversely, administration or overexpression of FGF19 in mice provided protection from diet-induced obesity and promoted the enhancement of energy expenditure as a result of increased hepatic fatty acid oxidation via suppression of acetyl-CoA carboxylase 2 and stearoyl-CoA desaturase 1, as well as an increased brown adipose tissue mass [94,102].

Recently reported observations have suggested the CNS to play a role in FGF19-mediated glucose homeostasis regulation, based on demonstrations of intra-cerebroventricular infusions of FGF19 improving glycemic status and potentiating peripheral insulin signaling in a murine insulin resistance model [103]. In addition, intra-cerebroventricular infusions of FGF19 also reduced both food intake and body weight [103,104].

Bile acid accumulation within hepatocytes can lead to mitochondrial dysfunction, endoplasmic reticulum stress, and immune cell infiltration, possibly producing inflammation, cell death, and hepatic injury [105,106,107]. In fact, NASH patients reportedly have elevated hepatic and circulating bile acid concentrations [108,109]. Furthermore, the circulating FGF19 concentration is reduced in patients with NAFLD/NASH [110,111], suggesting that dysregulated FGF19 expression might contribute to the pathogenesis of NAFLD/NASH. The aforementioned pleiotropic effects of FGF19 confirm the FGF19 agonism-inducing strategies to be promising therapeutic approaches for the treatment of NASH. However, the therapeutic promise held by FGF19 is blunted, to some degree, by its mitogenic potential which raises the possibility of hepatic tumorigenesis [112,113]. As this tumorigenic activity has been ascribed to FGFR4 [114,115], FGF19 variants specifically designed to eliminate FGFR4 binding have been generated. One engineered variant of FGF19 (NGM282, also known as M70) showed fully retained bile acid regulatory activity, but was devoid of pro-tumoral activity in mouse models [116]. NGM282 exhibited differential signaling pathway activation as compared with FGF19, activating only a subset of signaling pathways downstream from FGFR4. In animal models of NASH, treatment with NGM282 resulted in rapid and profound reductions in the concentrations of ALT and AST, as well as clear improvements in all histological features of NASH with suppression of *de novo* bile acid synthesis and inhibition of fatty acid synthesis and *de novo* lipogenesis, presumably through mechanisms that ameliorate hepatic bile acid toxicity and lipotoxicity [117].

**Figure 2:**
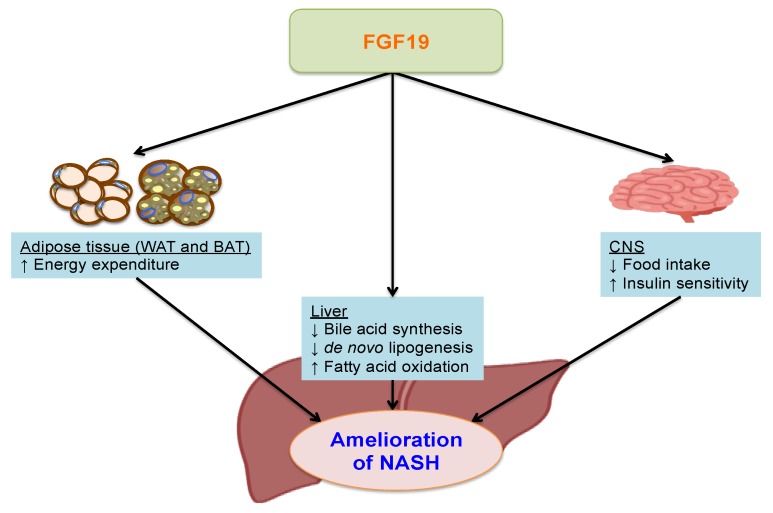
The putative beneficial effects of FGF19 on the pathogenesis of NASH. In the liver, fibroblast growth factor (FGF) 19 suppresses bile acid synthesis, increases fatty acid oxidation and suppresses de novo lipogenesis. In adipose tissue, elevated FGF19 in brown adipose tissue enhances energy expenditure, and in the central nervous system (CNS), FGF19 reduces food intake and improves insulin sensitivity. Overall, these pleiotropic effects of FGF19 ameliorate hepatic steatosis, bile acid toxicity and lipotoxicity, thereby exerting beneficial effects on the pathogenesis of non-alcoholic steatohepatitis (NASH).

In healthy individuals, NGM282 was safe and well tolerated and reduced serum concentrations of 7α-hydroxy-4-cholesten-3-one (C4), a marker for hepatic CYP7A1 activity [118]. Recently, in a randomized, placebo-controlled, double-blind study, NGM282 3 mg and 6 mg doses were shown to reduce C4 concentrations and significantly and rapidly improve the liver fat content over 12 weeks of treatment. In addition to changes in absolute liver fat content, NGM282 produced significant improvements in ALT, AST, and non-invasive serum fibrosis biomarkers including pro-peptide of type III collagen and the total enhanced liver fibrosis score. Patients in the 6 mg NGM282 group reportedly showed significant reductions in weight and BMI as compared to the placebo group, but the reductions in liver fat content were independent of weight loss and BMI. NGM282 was generally well tolerated at both doses, but adverse events including diarrhea, abdominal pain, and nausea were reported [119]. These results support further exploration of NGM282 for the treatment of NASH. Future studies should aim to elucidate optimal doses and the precise mechanisms of the anti-NASH effects of NGM282 in human subjects. The proposed beneficial effects of FGF19 against the pathogenesis of NASH are presented schematically in Figure 2.

## 6. Neurotensin

Neurotensin (NT) is a 13-amino acid peptide released from neuroendocrine cells of the small intestine [120]. NT exerts its physiological action by binding three NT receptor (NTR) types, NTR1, NTR2, and NTR3 [121,122]. The release of NT into the circulation is triggered by fat intake [123] and facilitates fatty acid translocation in the rat intestine [124]. In addition, NT increases small intestinal local blood flow [125] and pancreatic exocrine activity [126] promoting absorptive processes in the gut postprandially.

NT also functions as a neurotransmitter in the CNS by regulating pathways associated with ghrelin and leptin that mediate satiety and food ingestion in the lateral portion of the hypothalamus. In experimental mice, loss of the leptin action mediated by NT neurons co-expressing the long form of the receptor for leptin results in excess weight and impairs the ability to respond appropriately to energy deprivation, revealing NT to play a crucial role in mediating the leptin and ghrelin pathways [127]. These effects of NT are suggested to be involved in maintaining energy homeostasis and contributing to fat storage and metabolic disorders.

The fasting pro-NT (a stable NT precursor fragment produced in equimolar amounts relative to NT) concentrations were reported to be associated with the incidence of T2D, cardiovascular disease, breast cancer, and total and cardiovascular mortality [128]. NT deficient mice showed significantly reduced intestinal fat absorption and were protected from developing obesity, hepatic steatosis and insulin resistance associated with HFD feeding. Furthermore, NT was demonstrated to attenuate the activation of AMP-activated protein kinase and to stimulate fatty acid absorption in mice and in cultured intestinal cells. In human subjects, the same study also showed higher plasma pro-NT levels to be associated with obesity and insulin resistance, and doubled the risk of developing obesity later in life in non-obese subjects [129]. This study demonstrated a direct relationship between NT and increased intestinal fat absorption and obesity, and suggested that NT might be a prognostic marker of future obesity and a potential target for treating obesity-related diseases.

Recently, obese subjects with biopsy-proven NAFLD were shown to have significantly higher plasma pro-NT levels than those without NAFLD. Furthermore, the circulating pro-NT levels were shown to correlate positively with the presence and severity of NAFLD [130]. In morbidly obese patients, circulating pro-NT levels were also reported to be positively correlated with the presence and severity of NAFLD, as evaluated by the NAFLD activity score and histological evidence of visceral adipose tissue inflammation that leads to systemic low-grade inflammation, increased circulating fatty acid concentrations, insulin resistance and aberrant fat deposition in the liver [131]. These studies suggest that NT might be partially responsible for the pathogenesis of NAFLD/NASH through increased intestinal fat absorption and the induction of pro-inflammatory conditions in adipose tissue. Another report showed, however, that circulating levels of NT were decreased in women with NAFLD associated with morbid obesity as compared with to those in women of normal weight [132]. This discrepancy might be due to sex differences and variability in blood test results between NT (instable) and pro-NT (stable) [133]. The relationships between circulating NT/pro-NT levels in subjects with NAFLD/NASH are summarized in Table 2.

NT might be both a clinical parameter of and a therapeutic target for NAFLD/NASH, though larger studies with a longitudinal design are needed to elucidate the possible role of NT in the pathogenesis of NAFLD/NASH.

## 7. Conclusions

**Figure 3:**
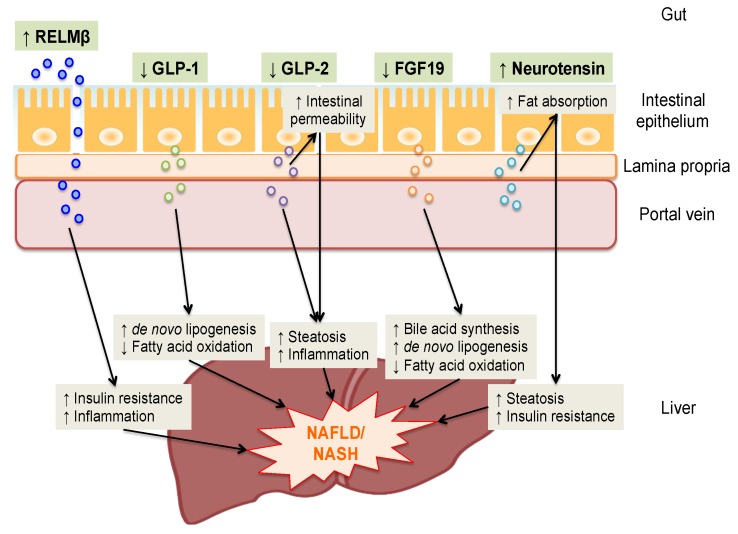
Summary of the putative roles of gut-derived secretory factors in the pathogenesis of NAFLD/NASH on the basis of the gut-liver axis. Resistin like molecule β (RELMβ) is secreted by goblet cells and delivered from the intestinal lumen into the lamina propria through a disrupted epithelial barrier, and then translocates into the portal vein and reaches the liver. Glucagon-like peptide-1 (GLP-1), glucagon-like peptide-2 (GLP-2), fibroblast growth factor (FGF) 19 and neurotensin are released from enteroendocrine cells into the lamina propria and reach the liver through the portal vein. Increased intestinal secretion and circulating RELMβ and neurotensin levels and decreases in those of GLP-1, GLP-2 and FGF19 could be involved in the pathogenesis of non-alcoholic fatty liver disease (NAFLD)/non-alcoholic steatohepatitis (NASH) in various ways.

The putative roles of gut-derived secretory factors in the pathogenesis of NAFLD/NASH on the basis of GLA are summarized in Figure 3. A growing body of evidence highlights the role of GLA in the pathogenesis of NAFLD/NASH. Thus, gut-derived secretory factors are considered to be a novel potential therapeutic target, and interventions such as GLP-1 analogues and recombinant variants of FGF19 have been utilized in clinical practice and examined in trials. Furthermore, other novel possibilities are currently being investigated. However, the precise mechanisms of action of gut-derived secretory factors in the pathogenesis of NAFLD/NASH have yet to be fully elucidated in humans. In conducting clinical studies, it is advantageous that secretory factors can be measured by blood test. Larger studies with a longitudinal design, and translational research are anticipated to provide additional insights into gut-derived secretory factors in the pathogenesis of NAFLD/NASH and may offer new biomarkers and therapeutic strategies.

## Figures and Tables

**Table 1:** Summary of clinical trials of GLP-1RA or DPP-4 inhibitors involving NAFLD/NASH patients.

References	Study Design	Study Subjects	Therapy and Follow-Up Duration	Outcomes
Cuthbertson et al. 2012 [46]	SA	25 (T2DM); NAFLD	Exenatide 20 μg (*n* = 19) or Liraglutide 1.2 mg (*n* = 6); 6 months	↓ALT; ↓Liver fat (^1^H MRS)
Armstrong et al. 2016 [47]	DB, RAND, PLAC	23; NASH (biopsy proven)	Liraglutide 1.8 mg vs. placebo; 12 months	Histology (disappearance of ballooning without worsening of fibrosis) improved
Eguchi et al. 2015 [48]	SA	19 (T2DM); NASH (biopsy proven)	Liraglutide 0.9 mg; 6 months	↓AST, ALT; ↓Liver fat (CT)
Cui et al. 2016 [52]	DB, RAND, PLAC	24 (prediabetes or early diabetes); NAFLD	Sitagliptin 100 mg; 6 months	Liver fat (MRI) not improved
Joy et al. 2017 [53]	DB, RAND, PLAC	6 (T2DM); NASH (biopsy proven)	Sitagliptin 100 mg; 6 months	Histology (Fibrosis and NAS) not improved

Abbreviations: ALT, alanine transaminase; AST, aspartate aminotransferase; DB, double blind; ^1^H MRS, proton magnetic resonance spectroscopy; NAFLD, non-alcoholic fatty liver disease; NAS, NAFLD activity score; NASH, non-alcoholic steatohepatitis; OAD, oral antidiabetic drug; PLAC; placebo controlled; RAND, randomized; SA, single arm; T2DM, type 2 diabetes mellitus.

**Table 2:** Summary of relationships between the circulating NT/pro-NT levels in patients with NAFLD/NASH.

References	Groups	Findings
Barchetta et al. 2018 [130]	28 Obesity without NAFLD; 32 Obesity with NAFLD	Obesity with NAFLD vs. Obesity without NAFLD, ↑Plasma pro-NT; Plasma pro-NT correlated positively with NAFLD, presence and severity
Barchetta et al. 2018 [131]	40 MO	Plasma pro-NT correlated positively with NAFLD presence and severity, and VAT inflammation.
Auguet et al. 2018 [132]	20 Normal weight; 18 MO without NAFLD; 33 MO with NAFLD	MO with NAFLD vs. Normal weight, ↓Plasma NT; MO with NAFLD vs. MO without NAFLD, ↓Plasma NT; No difference in plasma NT between SS and NASH.

Abbreviations: MO; morbid obesity; NAFLD, non-alcoholic fatty liver disease; NASH, non-alcoholic steatohepatitis; NT, neurotensin; SS, simple steatosis; VAT, visceral adipose tissue.

## Abbreviations

ALT	Alanine aminotransferase
AST	Aspartate aminotransferase
BMI	Body mass index
CNS	Central nervous system
DPP-4	Dipeptidyl peptidase-4
FXR	Farnesoid X receptor
FGF	Fibroblast growth factor
GLP-1	Glucagon-like peptide-1
GLP-2	Glucagon-like peptide 2
GLA	Gut-liver axis
HNF4α	Hepatocyte nuclear factor 4α
HFD	High fat diet
KO	Knock-out
LPS	Lipopolysaccharide
MCD	Methionine choline-deficient
NT	Neurotensin
NAFLD	Non-alcoholic fatty liver disease
NASH	Non-alcoholic steatohepatitis
RELMβ	Resistin like molecule β
SBS	Short bowel syndrome
T2DM	Type 2 diabetes mellitus
